# Tracheobronchoscopy and Oesophagoscopy

**Published:** 1908-12

**Authors:** Otis H. Johnson

**Affiliations:** Athens, Ga.


### TRACHEOBRONCHOSCOPY AND OESOPHAGOSCOPY.*

BY OTIS H. JOHNSON, B. A., M. D., ATHENS, GA.

Until quite recently direct inspection of the larynx and trachea has been possible only in a very limited and imperfect manner, by means of mirrors, and the removal of foreign bodies lodged in the lower portions of the trachea or oesphagus, or in the bronchial tubes, was well-nigh impossible without an incision. But, thanks to the genius of Professor Killain, of Freiburg, we now have, in his bronchoscope, an instrument by which we can in-

*Read before the Eighth District Medical Association of Georgia, at Athens, August 19, 1908.

spect every portion of the larynx, trachea, bronchi, and even *bronchioles,* the oesaphagus, and the greater part of the stomach, extract foreign bodies from these structures, make topical applications, or remove morbid growths. Formerly a foreign body in the bronchial tubes was almost certain to cause death eventually, unless coughed up, but now it is a comparatively simple matter to remove it through the long tube of the bronchoscope, by means of specially devised forceps.

Professor Killian's instrument, of which there are several modifications, conissts of a number of tubes of varying lengths and calibers, one of which is attached to a handle containing a miniature incandescent light, the rays from which are reflected through the tube by means of a fixed mirror, shielded from the eyes of the operator. The American bronchoscope, originally devised by Dr. Chevalier Jackson, and highly recommended by Kyle and Ballenger, differs from the German instrument in that the light is furnished by a tiny incandescent light at the distal end of the tube, instead of in the handle, and supplied by wires running through a conduit in the walls of the tube. The German type seems to be the more practical, as the light in the distal end of the Aemrican tube takes up valuable space, and is liable to be obscured by blood and secretions during the operation, while the light in the handle of the German tube cannot possibly be reached by secretions.

Bronchoscopy is usually required for the extraction of foreign bodies more than for any other purpose, though it is also used for the olcal application of weak solutions of silver nitrate in ulcerated and chronic conditions of the larynx and trachea, and for diagnostic purposes. It is practised upon children with as much success as upon adults, and is more often needed for them, since their universal habit of testing articles with the mouth brings about a large number of bronchial foreign body cases as a sequel. Also the danger is greater in the case of an infant, on account of the smaller size of the respiratory tubes, and the chance of immediate suffocation if not relieved.

A foreign body in the respiratory tract generally causes violent choking and suffocation at first, with cyanosis and aphonia, but these symptoms may subside, and not return for several hours or days. The cough may become constant, the patient loses weight, and there is frequently every symptom of pulmonary tuberculosis, except the presence of the bacillus.

According to Ballenger, "a history of spasmodic cough, dyspnoea and hoarseness, followed by a persistent cough, should excite suspicion of a foreign body in the respiratory tract if the patient is a small child."

Kyle and Johnson agree that "many curious cases of persistent cough and obscure bronchial or so-called lung trouble could be traced to some foreign body. The bronchial irritation or the persistent hacking cough may be due to the lodgement or some foreign body in the upper respiratory tract, or in the oesophagus."

No time should be lost in attempting to remove a foreign body from the respiratory passages, for at any moment it may assume a more unfavorable position and cause sudden death, or its long continued presence may cause local oedema, septic absorption, or pneumonia. If necessary, tracheotomy should be resorted to, in order to relieve the suffocative dyspnoea, though this will be useless if the foreign body is in the lower respiratory tract. The practice of thumping a person upon the back when choked, a method which is universally resorted to by the laiety, while sometimes successful, is nevertheless dangerous, for it may force the foreign body into a worse position and cause immediate suffocation. It is better to take the patient to a physician at once, without trying to remove it, and, if it cannot be removed by using laryngeal forceps and mirror, recourse should be had to the bronchoscope without delay.

There are two methods of bronchoscopy, *upper*, in which the tubes is introduced through the mouth, and *lower*, in which it is introduced through a tracheal incision. The former is practised in all cases, and the latter where the former has failed, a larger and shorter tube being used in the lower operation.

Upper bronchoscopy can be performed either under cocaine or with a general anaesthetic, ether being preferable to chlorform. The German operators prefer to pass the tube after a local application of cocaine, made first as a four per cent. spray, then as a twenty per cent. swab to the pharynx and larynx, and lastly, through the bronchoscope, as a ten per cent. swab to the trachea, but this can successfully be done only upon the stolid patient of a hospital clinic, and it will not do in private practice, nor especially upon children. In this method the patient is seated, his head thrown far back and seated by an assistant, the operator standing in front and over him.

With a general anaesthetic, the patient is only partially anaesthetized, in the dorsal position, and the head hangs over the end of the table, being supported by the assistant. Another assistant stands near with a bunch of long cotton swabs to remove secretions and cleanse the tube, or he may use Jackson's exhaust pump for removing saliva. The surgeon sits upon a low stool, or kneels upon the floor, at the patients head. A tube of the necessary length and caliber having been chosen, a mouthgag is inserted, and the tube passes down as far as the vocal cords, then held for an instant until it can be slipped between and past the cords during an inspiration. Now the trachea is swabbed, through the tube, with a ten per cent. solution of cocaine, to lessen reflex irritation, and the tube is slowly passed down the trachea, while the surgeon keeps a careful eye upon its course, until the trachael bifurcation is reached; then, if the right bronchus is to be explored, the tube is tilted to the left and passed on, and if the left is to be inspected, tilted to the right, the elasticity of the trachea and bronchi allowing considerable manipulation without damage. Thus the bronchioles may be examined in every direction, and the foreign body located. A pair of long-shaken forceps is introduced through the tube, and the offending object removed.

If the operator fails to find the foreign body, and thinks he can do so by lower bronchoscopy, or if he locates it and it is too large for removal by the upper route, he performs a low tracheotomy, introduces the tube through the wound and proceeds with the examination. This method is less likely to result in pulmonary complications, but is necessarily a more drastic procedure than upper bronchoscopy. The patient occupies the same dorsal position as in the upper operation, with the head hanging over the end and supported by an assistant. Strict asepsis must be observed in lower bronchoscopy, as in any other operation, and the wound must be well cleansed from blood before inserting the tube. After the operation a tracheotomy tube is kept in the wound for several days, and the patient is urged to sit up as soon as possible, to avoid pneumonia. The same instrument is used in examining the oesophagus, except that a mandrin or obturator must be used until the cricoid cartilage is passed, and the technique is practically the same as in bronchoscopy. Care must be taken, however, not to tilt the tube forward and compress the trachea..

Cases of a foreign body in the respiratory passage of oesophagus, which can be brought to the surgeon for bronchoscopic treatment are rather rare in private practice, and it is possible that the average specialist will never encounter a case; but every time a foreign body is extracted by the use of the bronchoscope a life is saved which would undoubtedly perish otherwise. Professor Killian has reported eighteen cases in which he attempted to remove a foreign body by this method, and in fifteen he was entirely successful. In two he failed to find the foreign body, and one other died of pulmonary complications six months after removal.

